# Predicting Prognosis of Early-Stage Non-Small Cell Lung Cancer Using the GeneFx® Lung Signature

**DOI:** 10.1371/currents.eogt.49eb4408c48237b8a8d4ed2060951083

**Published:** 2015-10-26

**Authors:** Sandy Der, Chang-Qi Zhu, Stacey Brower, Arlette Uihlein

**Affiliations:** Applied Molecular Oncology, Princess Margaret Cancer Centre, University of Toronto, Toronto, ON, Canada; Princess Margaret Cancer Center, University Health Network, University of Toronto, Toronto, ON, Canada; Helomics Corporation, Pittsburgh, PA, United States of America; Helomics Corporation, Pittsburgh, PA, United States of America

## Abstract

Use of adjuvant chemotherapy remains a complex decision in the treatment of early stage non-small cell lung cancer (NSCLC), with risk of recurrence being the primary indicator (i.e. adjuvant chemotherapy is considered for patients at high risk of recurrence but may not be beneficial for patients at low risk). However, although several clinical and pathological factors are typically considered when assessing the risk of recurrence, none are significantly associated with clinical outcome with the exception of tumor size. GeneFx® Lung (Helomics™ Corporation, Pittsburgh, PA) is a multi-gene RNA expression signature that classifies early stage NSCLC patients as high-risk or low-risk for disease recurrence. GeneFx Lung risk category has been shown to be significantly associated with overall survival in several independent clinical studies. The published literature regarding the analytical validity, clinical validity and clinical utility of GeneFx Lung is summarized herein.

## Clinical Scenarios

Despite successful surgery, 50-70% of early stage (stages I and II) non-small cell lung cancer (NSCLC) patients die within 5 years.^1^ Although the standard of care treatment for stage II patients, as well as high risk stage IB patients, is surgery followed by adjuvant chemotherapy,^2^ some stage II patients with a better prognosis may be spared the costs and adverse effects associated with chemotherapy. However, there are currently no reliable methods to identify these patients. There are 30-40% of stage I patients with a worse prognosis who may benefit from adjuvant treatment but methods to identify these patients are similarly lacking.^3^ Several clinicopathologic factors are currently used to estimate high risk of recurrence in NSCLC - poorly differentiated tumors, vascular invasion, wedge resection, tumors >4 cm, visceral pleural involvement, and incomplete lymph node samples. However, none of these factors (with the exception of tumor size) have been shown to be significantly associated with clinical outcome. Furthermore, as the current approach to oncology treatment moves toward stratified medicine, there is more focus on using the genetic composition of the tumor in individualizing patient treatment.^4^ As such, a prognostic marker for early stage NSCLC could have high clinical utility.

## Test Description

GeneFx® Lung is a 15 gene signature that can predict risk of recurrence in early stage NSCLC, independent of other, relevant clinicopathologic factors. RNA is isolated from NSCLC tissue, containing a minimum of 20% tumor which has been preserved in RNAlater® (an RNA stabilization reagent) or by being ‘fresh frozen’, using a phenol-chloroform method. Extracted RNA is reverse transcribed into cDNA and amplified using single primer isothermal amplification (SPIA) chemistry. The amplified cDNA is chemically and enzymatically fragmented and labelled with biotin. The cDNA targets are hybridized to an Affymetrix Human Genome U133 Plus 2.0 microarray, followed by a wash and stain procedure that binds streptavidin-phycoerythrin (SAPE) stain to the biotinylated cDNA molecules. Scanning identifies the number of cDNA transcripts that are present. The resulting data are processed using a proprietary algorithm to generate a risk score which is dichotomized into “high-risk” (risk score ≥ -0.10) or “low-risk” (risk score ˂ -0.10) categories. Patients whose tumors are categorized as high-risk should be considered, in concert with other clinical factors, for treatment with adjuvant chemotherapy.

GeneFx Lung distinguishes itself from other prognostic gene expression signatures for early stage NSCLC in a number of ways:

- GeneFx Lung is applicable to the two major histological subtypes of NSCLC, adenocarcinoma and squamous cell carcinoma.
- GeneFx Lung results are dichotomized into 2 categories (high-risk, low-risk), making results interpretation intuitive.
- GeneFx Lung is clinically validated across several independent patient cohorts.
- GeneFx Lung is clinically validated on multiple technology platforms (Affymetrix microarrays, Illumina and Agilent microarrays, RT-qPCR).
- GeneFx Lung shows promise as a predictive signature for response to adjuvant chemotherapy.^5^^,^ 6

## Public Health Importance

Lung cancer is the leading cause of cancer death. In 2014, it is estimated that there will be more than 225,000 new lung cancer cases in North America, 85% of which are NSCLC.^7^^,^ 8 ^,^ 9 Early stage NSCLC may be curable with surgical resection,^10^ and survival may be improved with adjuvant chemotherapy, especially in stage II patients.^11^^,^ 12 ^,^ 13 Thus, guidelines recommend that stage II NSCLC patients are treated with adjuvant chemotherapy^2^ although it is suspected that patients with a better prognosis (lower risk of recurrence) may be unnecessarily subjected to the costs and morbidity associated with chemotherapy with no clinical benefit. Likewise, guidelines recommend that stage I NSCLC patients are not to be treated with adjuvant chemotherapy,^2^ but a recurrence rate of 35-50% in this group^9^ suggests that adjuvant chemotherapy may be beneficial to the portion of stage I patients with a poorer prognosis (higher risk of recurrence). Currently, aside from tumor size, there are no clinically validated markers to discern prognosis and potential chemotherapy benefit in early stage NSCLC that may assist in developing a personalized treatment approach for these patients.

## Published Reviews, Recommendations and Guidelines

*Recommendations by independent groups*

The Wadsworth Center of the State of New York performed an independent review of the technology, standard operating procedures, quality measures, and analytical and clinical validation results of GeneFx Lung, resulting in full approval and licensure in the state of New York. GeneFx Lung testing is performed exclusively in the Helomics™ Corporation laboratory in Pittsburgh, Pennsylvania, which is certified to comply with the Centers for Medicare & Medicaid Services Clinical Laboratory Improvement Amendments (CLIA) program. The Helomics laboratory is licensed by CLIA, with independent licenses in the states of New York, California, Florida, Maryland, Pennsylvania and Rhode Island.

*Guidelines by professional groups*

The American Society of Clinical Oncology (ASCO) and The Cancer Care Ontario Program in Evidence-Based Care (CCO) jointly reviewed and provided recommendations regarding the role of adjuvant chemotherapy and radiation therapy in the treatment of patients with early stage NSCLC.^14^ Due to the lack of clinical evidence regarding the role of adjuvant chemotherapy, specifically in stage I disease, the recommendations do not support use of chemotherapy in this setting while still recognizing that high-risk patients could benefit from its use. These recommendations were generated prior to the advent of genomic signatures for prognosis of early stage NSCLC and, thus, do not mention use of this technology to discern high-risk patients.

*Independent review articles*

In 2009, Zhu et al. reviewed the concepts and methodologies involved in identifying, developing and validating multi-gene signatures in lung cancer.^15^ In addition to describing the variety of approaches to these processes, this review summarized a number of prognostic signatures independently validated in NSCLC, underscoring the strong clinical need for better prediction of patient prognosis in this disease. Similar ‘guidelines’ for developing and validating prognostic signatures in NSCLC were described by Subramanian and Simon.^16^ More recently, a variety of NSCLC prognostic markers (including single gene, immunohistochemical and multi-gene makers) were reviewed, suggesting the potential value of such markers in predicting benefit from adjuvant chemotherapy in early stage NSCLC.^17^

## Evidence Overview

*Analytical Validity*

- The precision, sensitivity and specificity of GeneFx Lung were recently evaluated, confirming the robust and reliable nature of the test.^18^ Specifically, agreement of 97% or greater amongst numerous replicates of the assay both between runs and within the same run reveal the highly repeatable and reproducible nature of GeneFx Lung. In addition, the lower limit of quantitation was established, and genomic DNA was found to not interfere with assay results.
- The accuracy of GeneFx Lung was evaluated in a study of 34 NSCLC samples in which biological replicates were assayed in the commercial laboratory (Helomics™ Corporation, Pittsburgh, PA, USA) as well as an accredited reference laboratory (Almac Diagnostics Ltd., Craigavon, Northern Ireland, UK).^18^ The concordance in risk categorization between the two laboratories was 94% (95% CI 86%-100%), with a Pearson correlation of 0.88 (95% CI 0.77-0.94). This level of concordance is deemed to be acceptable as it exceeds the level of concordance observed within the commercial laboratory (see next bullet point). Furthermore, there was no evidence of bias between the laboratories.
- Although originally developed and validated using fresh frozen tissue, GeneFx Lung has been shown to perform equivalently in tissue preserved in RNAlater.^18^ In a study of matched fresh frozen and RNAlater-preserved NSCLC tissue from 43 patients, the percent concordance in risk category between the tissue formats was 84% (95% CI 73%-95%), with a Pearson correlation of 0.74 (95% CI 0.63-0.85) of the risk scores. The level of agreement observed between matched fresh frozen and RNAlater-preserved tissues is comparable with the inherent reproducibility observed within biological replicates of fresh frozen tissue (79% concordance, 0.83 Pearson correlation).
- GeneFx Lung maintains performance across various gene expression technology platforms. Although developed and validated on the Affymetrix U133 microarray platform, GeneFx Lung maintained statistical significance when using the Agilent 44K platform [hazard ratio (HR) 2.27, 95% CI 1.18-4.35, p=0.014] as well as RT-qPCR (HR 2.29, 95% CI 1.06-4.94, p=0.034).^5^

*Clinical Validity*

- In a prospective evaluation of 181 untreated early stage NSCLC patients, GeneFx Lung-designated risk of recurrence (high-risk vs. low-risk) was significantly correlated with overall survival, with a multivariate HR of 1.95 (95% CI 1.15-3.30, p=0.013). The prognostic ability of GeneFx Lung was corroborated in subgroup analysis for stage I (HR 2.17, 95% CI 1.12-4.20, p=0.018) and stage IA (HR 5.61, 95% CI 1.19-26.45, p=0.014) patients, with 92% of low-risk, but only 61% of high-risk, stage IA patients achieving survival at 5 years. Furthermore, the signature’s prognostic ability is independent of histology, with utility in both adenocarcinoma and squamous cell carcinoma cases.^6^
- Independent, published microarray datasets were used to validate the prognostic property of GeneFx Lung, which had been derived from 62 untreated early stage NSCLC patients in the JBR.10 clinical trial. GeneFx Lung was shown to be a significant prognostic factor (independent of other clinicopathological factors) in three datasets totaling 308 early stage NSCLC patients, with HRs of 2.26, 2.27 and 3.57, spanning both adenocarcinoma and squamous cell carcinoma, as well as other less common histological subtypes.^5^

*Clinical Utility and Other Supportive Studies*

- Initial studies have suggested that the GeneFx Lung signature may also be predictive of improved overall survival in early stage NSCLC patients treated with adjuvant chemotherapy. In two separate datasets, patients designated as high-risk by GeneFx Lung and treated with adjuvant chemotherapy experienced significantly improved overall survival compared to those left untreated.^5^^,^ 6 Alternatively, adjuvant chemotherapy in low-risk patients may not be beneficial.^5^

## Limitations

GeneFx Lung has been shown to perform equivalently in both fresh frozen and RNAlater-preserved tissue formats, however, performance in formalin-fixed paraffin embedded (FFPE) tissue has yet to be explored. FFPE represents a more stable and clinically accessible tissue format for clinical studies as well as commercial use.

Clinical validations performed to date clearly indicate the prognostic significance of GeneFx Lung in designating the risk of recurrence in early stage NSCLC tumors. Because risk of recurrence may play a large role in making treatment decisions, the predictive significance of the signature should also be considered. Exploratory analyses have been promising, and additional, independent validations of GeneFx Lung as a predictive marker are warranted and should consider use of tumor recurrence-based endpoints (e.g. disease free survival).

## Conclusions

Although numerous factors are considered when staging NSCLC, there remains a clinical unmet need to delineate risk of recurrence in early stage patients as stage alone does not fully elucidate which patients may benefit from adjuvant chemotherapy. Clinicopathological factors currently employed are not significantly associated with treatment effectiveness, with the exception of tumor size. GeneFx Lung is a robust gene signature that has been validated in several independent cohorts to estimate risk of recurrence in early stage NSCLC, with overall survival being significantly associated with GeneFx Lung risk category. The studies reviewed herein support use of GeneFx Lung to assess risk of recurrence in both adenocarcinoma and squamous cell carcinoma cases of early stage NSCLC, thereby facilitating adjuvant chemotherapy treatment decisions in these patients.

## Competing Interests

SB and AU are paid employees of Helomics Corporation and hold stock options. These competing interests do not affect our adherence to the PLOS Currents policies on sharing data and materials.
